# Real-Time PCR Protocol for Detection and Quantification of Three Pathogenic Members of the Vibrionaceae Family

**DOI:** 10.3390/microorganisms10102060

**Published:** 2022-10-18

**Authors:** Cátia Costa, Guilherme D. Ferreira, Marco Simões, Joana L. Silva, Maria J. Campos

**Affiliations:** 1 MARE-Marine and Environmental Sciences Centre, 2520-630 Peniche, Portugal; 2 MARE-Marine and Environmental Sciences Centre, ESTM, Polytechnic of Leiria, 2520-630 Peniche, Portugal; 3 Allmicroalgae Natural Products S.A., Rua 25 de Abril, s/n, 2445-413 Pataias Gare, Portugal

**Keywords:** qPCR, *Vibrio alginolyticus*, *Listonella anguillara*, *Vibrio harveyi*, vibriosis, aquaculture

## Abstract

Vibriosis, an often-fatal disease induced by pathogenic members of the Vibrionaceae family, causes severe economic losses in aquacultures. To mitigate/avoid vibriosis outbursts, it is vital to detect and quantify these pathogens as early as possible. However, standard microbiological methods are time-consuming and often underestimate cell counts, which calls for the development of valid alternatives. In this study, real-time polymerase chain reaction (qPCR) was employed to detect the pathogenic species *Vibrio alginolyticus*, *Listonella anguillara*, and *Vibrio harveyi* using a new primer pair targeting the *groEL* gene. In addition, the DNA extraction efficiency of three methods, two commercial kits and the boiling method, was compared. The most efficient method was the DNeasy Blood and Tissue kit, with a detection limit ranging between 154 and 600 CFU mL^−1^ in the case of *V. alginolyticus* and *L. anguillara*, and 48 CFU mL^−1^ for *V. harveyi*. Thus, this study presents the development and evaluation of a method for the early quantification of all three species in saline suspensions. However, the results obtained by spiking a microalgae sample with *V. harveyi* emphasize the importance of adjusting the DNA control’s standard curve to the relevant extraction matrices, as it affects the DNA extraction efficiency and may hamper an accurate quantification with qPCR.

## 1. Introduction

The genus *Vibrio* comprises more than 130 species registered on the List of Procaryotic Names with Standing Nomenclature [1] with a detailed description and valid nomenclature. *Vibrio* spp. are Gram-negative motile bacteria, facultative anaerobic, non-spore-forming, and have the shape of a curved or straight rod [2,3]. Vibrios are ubiquitous in aquatic environments and play an important role in the degradation of organic matter [4], on a seasonal basis [5]. Aside from their important ecologic role, some species of this genus are opportunistic pathogens and have been associated with illnesses in humans and marine animals [6].

The ingestion of undercooked seafood is the most common reason for illness in humans caused by *Vibrio* spp., the most hazardous pathogens being *Vibrio vulnificus*, *Vibrio parahaemolyticus*, and *Vibrio cholerae* [7,8]. The first causes wound infections and septicaemia [9], the second is responsible for gastroenteritis [8], and the last is responsible for cholera, one of the main causes of death in developing countries [10]. The most prevalent bacterial disease in diverse marine fish and molluscs is vibriosis, which is loosely defined as haemorrhagic septicaemia after vibrio infection [2]. Oftentimes, particularly in early life stages, vibriosis causes massive death in aquacultures [11,12,13,14].

Some of the most common pathogens of marine species include *Listonella anguillara* [15,16] (formerly known as *Vibrio anguillarum* or *L. anguillarum*), *Vibrio harveyi* [17,18], and *Vibrio alginolyticus* [19,20]. Altogether, their economic impact on aquaculture is huge, which implies the need to control and prevent outbursts of these pathogenic agents [21]. There are a few strategies to keep aquacultures safe and diseases controlled, such as good farming practices like sanitation [22]. In addition, the spread of infections can be controlled using antibiotics, topical disinfectants, or by increasing the abundance of microalgae [22,23,24]. Enhancing the immune defence system of fish is also important to overcome this problem, through vaccination for instance [25].

Conventional culturing methods can be used to detect and quantify pathogenic members of the Vibrionaceae family, despite being extremely time-consuming and laborious [26]. Furthermore, direct colony counting in thiosulfate-citrate-bile salts-sucrose agar (TCBS—specific media for members of the Vibrionaceae family) is typically underestimated [27]. Unsurprisingly, molecular approaches have been employed to detect and quantify bacteria [28,29,30], avoiding the possible bias and labour-intensive cultivation [31]. Indeed, the polymerase chain reaction (PCR) method provides a rapid, specific, and sensitive analysis of the target sequence, which can be genus- or even species-specific [32,33]. However, PCR does not enable a direct quantification of the target sequence [34,35,36]. On the other hand, real-time PCR (qPCR) is faster than the conventional PCR, since it does not need an electrophoresis step, and can be used to quantify microorganisms [28,29,30]. Additionally, the use of qPCR for diagnosing assays has increased its popularity, mainly due to its speed, better sensitivity, specificity, and reduced risk of carryover contamination compared to conventional diagnostic methods [37,38]. In this regard, it seems like the detection and quantification of pathogenic *Vibrio* spp. for marine species should follow the footsteps of its human-pathogen counterparts (such as *V. parahaemolyticus* [39,40], *V. cholerae* [41], or *V. vulnificus* [42]) and embark on the molecular era. Despite all of the advantages of qPCR, one major drawback is the fact that this technique is unable to distinguish live and dead bacteria compared to the conventional culturing methods [43].

Not all genes are suitable for species identification, as gene mutation rates are often uncoupled from species diversification rates. For bacteria, the 16S rRNA gene is frequently used for this purpose [44]. However, in the specific case of the Vibrionaceae family, many closely related species share a high degree of sequence similarity in the 16S rRNA gene [45], which reduces the utility of this gene for species discrimination in this group of bacteria. The *groEL* gene (also known as HSP60) encodes a molecular chaperon, is extremely conserved in nature [46], and is more heterogeneous than the 16S rRNA gene. Therefore, *groEL* sequences have a large degree of interspecies variation [47]. Thus, this gene is a better candidate for the identification of species from the Vibrionaceae family and has already been used to identify a few isolates [33,48,49].

The main objective of this work was to develop an effective qPCR protocol to detect and quantify three pathogenic members of the Vibrionaceae family known to affect marine aquacultures. The species that were chosen were *L. anguillara*, *V. harveyi*, and *V. alginolyticus* and *groEL* was the chosen gene. This protocol could, ideally, be used preventively by aquaculture producers to avoid pathogenic outbursts of Vibrionaceae, by detecting them in advance. However, it must be clarified that, in this study, the qPCR protocol was designed using a saline solution matrix. It should be possible to adapt this protocol to aquaculture waters, but it is important to acknowledge the possible presence of contaminants or inhibitors that could possibly affect extraction or the qPCR reaction and thus the protocol. In addition, tests were made using a complex matrix (microalgae culture) spiked with known *V. harveyi* concentrations to determine whether the current protocol was directly applicable to other sectors or if, on the other hand, a different extraction matrix affected the DNA extraction efficiency.

## 2. Materials and Methods

### 2.1. Absolute Microbial Enumeration

For absolute microbial enumeration, *V. alginolyticus* NCIMB 1903, *L. anguillara* NCIMB 6, and *V. harveyi* NCIMB 1280 were bought from Colección Española de Cultivos Tipo (CECT). The first was grown in TCBS (VWR, Radnor, PA, USA) because of its swarming capability in non-selective media [50], incubated for 24 h at 28 °C. The second was grown in tryptic soy agar (Biolife, Milano, Italy) and incubated for 24 h at 28 °C, while the third was grown in nutrient agar (VWR, Radnor, PA, USA) and incubated for 24 h at 26 °C as suggested by CECT. Colony-forming units (CFU) per mL of microbial suspension were determined by counting the surface plating on media. Each species was suspended in 0.85% (*w*/*v*) saline solution and adjusted to the 0.5 McFarland turbidity standard on a densitometer (VWR, Radnor, PA, USA). Then, 10-fold serial dilutions up to 10^−^^6^ were made in saline solution (0.85% *w*/*v*) and inoculated in triplicate for counting. This procedure was repeated three times to obtain three independent replicates for each species. Simultaneously, from each dilution and each replicate, 3 mL were sampled to address the effects of different DNA extraction methods (1 mL per method; detailed in the next section) and centrifuged at 10,000× *g* for 10 min for the collection of the pellets.

### 2.2. DNA Extraction Methods

For DNA extraction of the bacterial pellets, three different methods were used. One method was a commercial kit based in chloroform extraction, Gen kit (Thermo Fisher Scientific, Waltham, MA, USA). The second method, DNeasy kit (Qiagen, Hilden, Germany), contains silica columns for DNA purification. The third and final method was the boiling method [51]. Both Gen kit and DNeasy kit protocols were followed according to the manufacturer’s instructions. For the boiling method, the bacterial pellets were resuspended in 100 µL of sterile Milli Q water and heated at 100 °C for 10 min, followed by centrifugation at 10,000× *g* for 5 min for collection of the supernatant. After conducting all extraction methods, the DNA solutions were kept at −20 °C until further use.

### 2.3. Primer Pair Design and PCR Conditions Optimisation

For the design of the primers, the *groEL* gene sequences of 14 species belonging to the Vibrionaceae family and 4 outgroup species (available on National Center for Biotechnology Information, NCBI) were aligned by Clustal W using the MegAlign software (DNASTAR Inc., Madision, WI, USA). The accession numbers can be found in Table 1 and the alignment depicting the zone for primer annealing can be found in Figure S1. The primers were designed in a conserved zone of the Vibrionaceae family DNA sequences with forward primer sequence being 5′-ATCACTGTTGAAGAAGGTCAA-3′ (groEL200_FW) and the reverse primer sequence being 5′-ATGATCAGTAGTGGGCGAGA-3′ (groEL200_REV), resulting in a fragment of ca. 200 bp (*V. alginolyticus—*206 bp; *L. anguillara* and *V. harveyi—*227 bp). The G+C content for the forward and reverse primers is 38.10% and 50%, respectively. The specificity of primers was verified by using the Basic Local Alignment Search Tool (BLAST^®^; https://blast.ncbi.nlm.nih.gov/Blast.cgi, accessed on 14 February 2022) against the publicly available sequences.

To ensure primer specificity against the target microorganisms, and to determine the optimal annealing temperature, DNA from *V. alginolyticus*, *L. anguillara*, and *V. harveyi* was used as the template, as well as the DNA of *Escherichia coli*, *Salmonella enterica*, *Yersinia ruckeri*, *Aeromonas hydrophila*, and *Klebsiella pneumoniae*. The DNA template for the PCR reaction was obtained by the boiling method. The PCR reactions consisted of NZYTaq II 2x Green Master Mix (Nzytech, Lisbon, Portugal), primers (0.5 μM) and 5 μL of DNA, for a total volume of 20 μL. The PCR was performed in a T100 thermal cycler (BioRad, Hercules, CA, USA), and the conditions were the following: 1 cycle of denaturation at 95 °C for 5 min, followed by 35 cycles of denaturation at 95 °C for 30 s, an annealing step between 63–67 °C for 30 s, an elongation step at 72 °C for 40 s, and a final cycle of elongation at 72 °C for 5 min. The optimal annealing temperature that resulted in the sole amplification of the three target species was 63 °C.

### 2.4. Absolute qPCR Calibration Curves

The qPCR assays were conducted on a CFX ConnectTM Real-Time PCR detection system (BioRad, Hercules, CA, USA). Each reaction was made in triplicate for a final volume of 10 µL including 5 µL of iTaq™ Universal SYBR^®^ Green Supermix (Bio-Rad, Hercules, CA, USA), 0.8 µM of forward and reverse primers, and 1 µL of template DNA and sterile Milli-Q water to make up the final volume. The qPCR conditions were set as 95 °C for 3 min (hot-start), followed by 50 cycles of 95 °C for 20 s (denaturation step), and 63 °C for 45 s (annealing/extension) step. The number of cycles resulted from an optimization of qPCR conditions, as preliminary results using 40 cycles failed to detect the most diluted samples of *V. alginolyticus*. The DNA template from the three species (*V. alginolitycus*, *L. anguillara*, and *V. harveyi*) was obtained from the three DNA extraction methods that have already been described.

The quantification cycle (Cq) value corresponds to the qPCR cycle number where the fluorescence rises above the baseline threshold [52]. The baseline threshold was manually set to 160 relative fluorescent units (RFU) since it was the lowest possible value that proved to be adequate for the detection of all reactions. To estimate qPCR efficiency (*E*, %), the Cq must be plotted versus the logarithm of the absolute bacterial concentration (CFU mL^−^^1^) in order to determine the slope (*m*) of the linear regression fit, followed by the application of Equation (1) [53].

(1) $$E=\left(-1+10^{-\frac{1}{m}}\right)\times 100$$

### 2.5. The Influence of a Complex Matrix in Quantification of Artificially Spiked Microalgae Samples

*Nannochloropsis oceanica* samples were used as a matrix for spiking with known concentrations of *V. harveyi*. The microalgae samples were tested before with a PCR using the groEL200 primer pair to ensure that no *Vibrio* spp. were present. Samples from *N. oceanica* (270 µL) were spiked with 30 µL of known *V. harveyi* concentrations (CFU mL^−^^1^) and the DNA was extracted using the DNeasy kit.

## 3. Results

### 3.1. The Standard Curves

**Figure 1:**
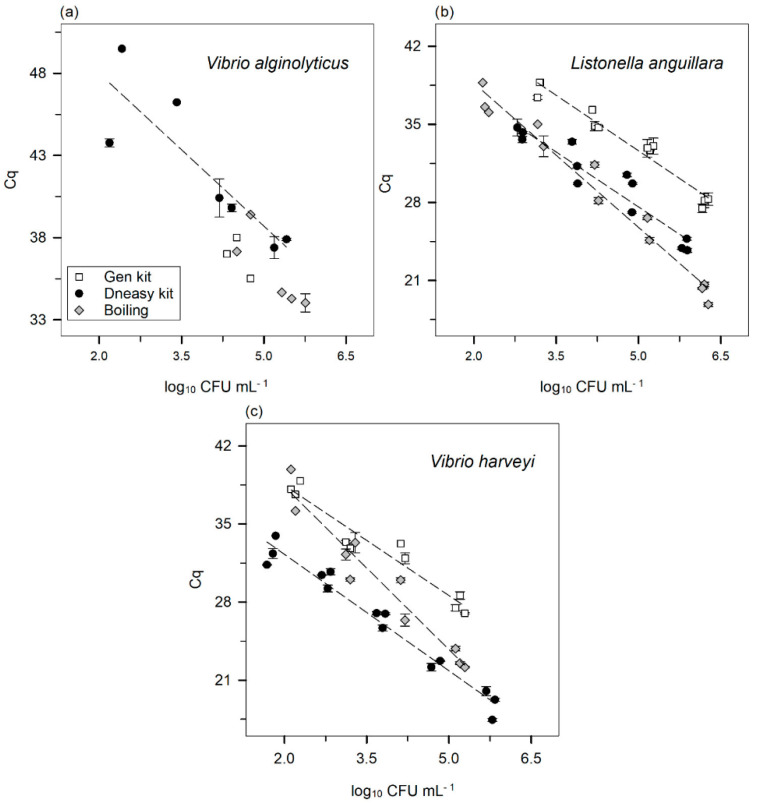
Standard curves correlating the logarithm of CFU mL^−1^ of bacterial suspension with Cq for (**a**) *V. alginolyticus*, (**b**) *L. anguillara*, and (**c**) *V. harveyi*. The three DNA extraction methods are depicted with different symbols: 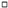
—Gen kit, 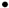
—DNeasy kit, and 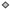
—boiling method. Error bars ± standard error. The Cq was always obtained with a threshold of 160 RFU.

The standard curves using 10-fold dilutions (five concentrations) of DNA obtained from pure cultures of the three target species and extracted by three independent methods are shown in Figure 1. The standard curves correlate the CFU mL^−1^ of bacterial suspension with the Cq (obtained with a threshold of 160 RFU).

When using DNA obtained with the Genomic DNA Purification kit (Gen kit), it was not possible to obtain a standard curve for *V. alginolyticus* since the Cq was only determined in one microbial concentration (Figure 1a). The same was observed with DNA extracted with the boiling method for the same species, where only two concentrations yielded a Cq. Accordingly, it was only possible to draw one standard curve for *V. alginolyticus*, with DNA extracted using the DNeasy Blood and Tissue kit (DNeasy kit). For *L. anguillara* (Figure 1b) and *V. harveyi* (Figure 1c), the three DNA extraction methods allowed the determination of a Cq value in at least four bacterial concentrations. The detection limit is the minimum number of CFU that the qPCR can detect, i.e., the highest Cq value (lowest CFU mL^−1^ count) corresponds to the detection limit. The detection limits using the DNeasy kit were, for *V. alginolyticus* (Figure 1a) and *L. anguillara* (Figure 1b), ca. 154 and 620 CFU mL^−1^, respectively. In the case of *V. harveyi* (Figure 1c), the detection limit was lower (48 CFU mL^−1^). Using the Gen kit, the detection limits were much higher than the ones obtained with the Dneasy kit. For *V. alginolyticus*, it was ca. 2.1 × 10^4^ CFU mL^−1^ (Figure 1a), ca. 1.4 × 10^3^ CFU mL^−1^ for *V. anguillarum* (Figure 1b), and 131 CFU mL^−1^ for *V. harveyi* (Figure 1c). While using the boiling method, the detection limits were higher in the case of *V. alginolyticus* (Figure 1a) and *V. harveyi* (Figure 1c)*,* ca. 3.2 × 10^4^ and 131 CFU mL^−1^, respectively. In the case of *V. anguillarum*, it was 143 CFU mL^−1^ (Figure 1b).

### 3.2. The qPCR Efficiency (E%)

The qPCR efficiency (*E*, %) was determined using the slope from the equations derived from the standard curves and applying Equation (1) (Table 2).

The *E* ranged between 60 and 110%, being the highest value from *V. alginolyticus*. The R^2^ from the linear regression of *V. alginolyticus* was 0.76, while the R^2^ for the other two species was higher (0.94, on average).

**Figure 2:**
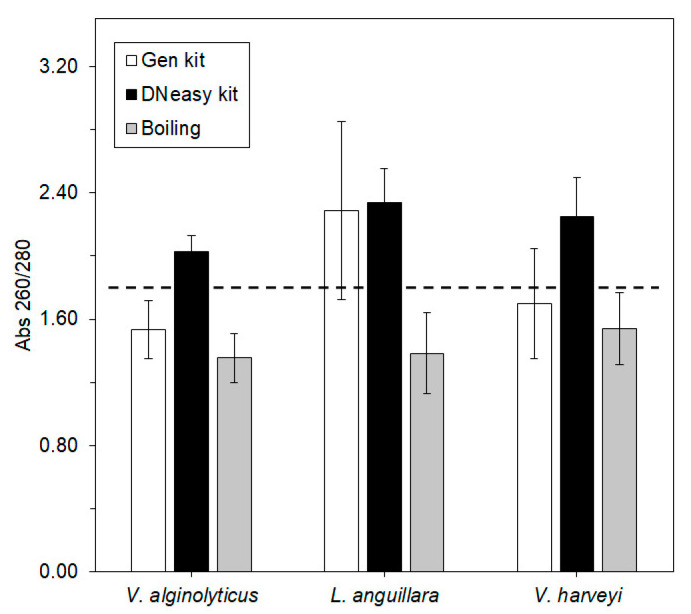
Purity of extracted DNA of *V. alginolyticus*, *L. anguillara*, and *V. harveyi* using three DNA extraction methods assessed by the ratio of absorbances at 260 and 280 nm: 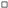
—Gen kit, 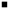
—DNeasy kit, and 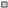
—boiling. The dashed line denotes the threshold ratio of 1.80, above which DNA is considered pure. Error bars ± standard error.

The purity of the extracted DNA by the three methods was assessed by the ratio of absorbances at 260 and 280 nm (Figure 2).

The absorbances from the DNA extracted with the DNeasy kit were higher than 1.80 in all three species. The absorbances from DNA extracted by the boiling method were under the threshold of 1.80. In the case of the Gen kit, the absorbance was higher than 1.80 only in *L. anguillara*, being lower than this value in the remaining species.

### 3.3. The Influence of Complex Matrices

**Figure 3:**
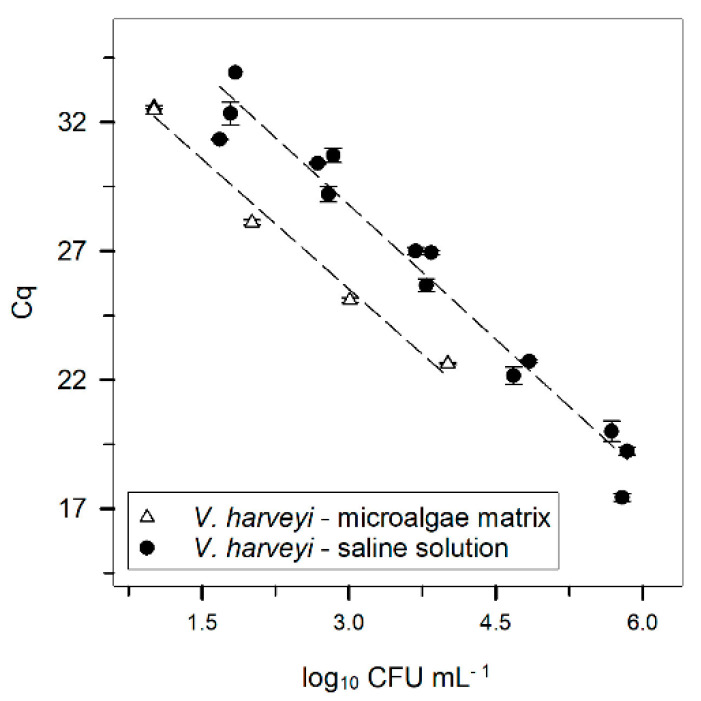
Correlation between the logarithm of *V. harveyi* (CFU mL^−1^) concentration in different matrixes—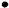 saline solution (data as shown in Figure 1), and 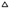 microalgae matrix. Both results were obtained after DNA extraction using the DNeasy kit. Error bars ± standard error. The Cq was always obtained with a threshold of 160 RFU.

To test the influence of a complex matrix in the DNA extraction step of the protocol, a microalgae culture of *N. oceanica* was spiked with known concentrations of *V. harveyi*, and the results compared with the standard curve obtained with the DNeasy kit method for *V. harveyi* (Figure 3). For the same *V. harveyi* concentration, the Cq was lower with the microalgae matrix than in the saline solution matrix.

## 4. Discussion

The DNA extraction is a crucial step across molecular methods because, if neglected, leads to an underestimation of the true number of microorganisms present in the sample, becoming a source of systematic error [52,54]. In optimal conditions, the extraction yield from every single cell in a sample should be 100%. From the three DNA extraction methods used in this study, the Gen kit was the least efficient on all three species, as seen by a higher Cq for the same bacterial concentration with respect to the other two methods (Figure 1). In the case of *V. alginolyticus* (Figure 1a), it was not even possible to fit an accurate standard curve to the data using this method.

Some studies compare different DNA extraction methods and report that the DNA extraction efficiency of the boiling method is not different from the one achieved with commercial kits [55,56,57]. In this study, however, the boiling method exhibited similar results only in comparison with the DNeasy kit, and only in the species *L. anguillara* (Figure 1b). For example, for a bacterial concentration of 1 × 10^5^ CFU mL^−1^, the resulting Cq with the boiling method was 25.76 and 27.60 with the DNeasy kit. For *V. harveyi* (Figure 1c), the DNA extraction efficiency using the boiling method was lower than using the DNeasy kit, i.e., with the same example concentration, the Cq for the boiling method was 23.73, whereas for the DNeasy kit, it was 21.84.

Regarding qPCR efficiencies (*E*; Table 2), the boiling method resulted in much lower values than those obtained with the commercial kits, irrespective of the species. It is important to mention that this comparison is impossible in *V. alginolyticus* due to the lack of DNA extracted with the boiling method. In the case of *L. anguillara* and *V. harveyi*, the boiling method resulted in an *E* of 71% and 60%, respectively, whereas using kits, these values were close to 100% (Table 2). Theoretical qPCR efficiencies should range between 0 and 1 (i.e., 0–100%), though likely range between 90–105% [58].

There are several reasons for a lower qPCR efficiency which include, for example, bad primer design, the presence of PCR inhibitors, or cross contaminations [42,52]. It is known that the boiling method yields impure bacterial DNA (260/280 ratio of ca. 1.4—[59]), which could explain the low qPCR efficiency due to the possible carryover of proteins. Indeed, the analysis of Figure 2 suggests the presence of contaminants in the extracted DNA for all species when the boiling method was chosen. The purity of the DNA extracted using both kits was always higher than with the boiling method. In the case of *V. alginolyticus*, the low *E* (Table 2) does not appear to be completely explained by the purity of the DNA (Figure 2). The groEL200 primers are not a perfect match to the *groEL* gene sequence of *V. alginolyticus* (three nucleotide mismatches in the forward primer and three in the reverse—Figure S1 of the Supplementary Information). Despite having a forward primer with a G+C content slightly lower than the recommended (between 40–60% [60,61]), the reverse primer displays a high G+C content at the 3′ end [62], which compensates the difference. Nevertheless, it was confirmed that the groEL200 primers were able to amplify DNA from *V. alginolyticus* (Figure S2). Overall, the results achieved using the Gen kit and the boiling method were not as good as those achieved with the DNeasy kit, either due to a lower DNA extraction efficiency or lower qPCR efficiency. Accordingly, from the data gathered in the present study, the use of the DNeasy kit is recommended, irrespective of the target species.

One other aspect that was addressed in this study was whether the proposed protocol could be directly applicable to other sectors which may benefit from the detection and quantification of pathogenic members of the Vibrionaceae family. One such sector is the production of microalgae, as these may act as vectors for the transmission of vibriosis if these bacteria are present in the cultures prior to the incorporation in aquaculture feeds. Therefore, serial dilutions of *V. harveyi* were tested for the quantification of spiked microalgae cultures (Figure 3). As the Cq from one bacterial concentration in a microalgae matrix is lower than the Cq obtained for the same bacterial concentration in saline solution (simple matrix, i.e., water), it seems that, at first sight, having a different matrix influences the DNA extraction efficiency. In general, when DNA is extracted from complex matrices (e.g., faeces, food, or other samples), the efficiency of DNA recovery is ca. 30% or lower [63,64,65]. Indeed, regarding the studies of Blackstone et al. [66] and Kaufman et al. [67] with *V. parahaemolyticus*, the presence of oyster tissue decreases the DNA extraction efficiency. However, this trend is not unique since the opposite (i.e., better DNA extraction efficiency in a complex matrix) has already been reported [26]. In spite of increasing or decreasing DNA extraction efficiency, it is clear that different extraction matrices require the development of different standard curves, as previously suggested by Prol et al. [26] and confirmed by this study. Accordingly, the standard curves presented in this work for the quantification of the three species are valid for similar matrices. Thus, waters from aquaculture tanks could be analysed by the methods shown here and inform the producers in advance if a pathogenic member of the Vibrionaceae family is present and at what concentration.

Another hypothesis that could justify the differences observed in the microalgae and saline solution matrices concerns the total extraction volume. In this study, the standard curves were obtained using 1 mL of bacterial suspension in saline solution; however, only 300 µL of microalgae suspension (270 µL of microalgae + 30 µL bacterial suspension) were used to test the accuracy of the standard curves. Campbell and Wright [42] state that decreasing the volume of a sample may increase the recovery of DNA after the extraction procedure, since it improves cell lysis. Thus, the smaller volumes used with the microalgae matrix could have increased the DNA extraction efficiency of the method. However, this hypothesis cannot be confirmed since larger volumes in the microalgae suspension were not assessed in this study. Nevertheless, it is important to mention that the DNeasy kit has been developed for the analysis of soil and these kits are used frequently to obtain DNA from microalgae cultures [68,69,70], which could explain the greater DNA extraction efficiency in this matrix.

The accuracy of microbial quantification with qPCR is related to the value of trueness (degree of agreement of the expected value with the true value or accepted value) [52]. There are no fixed values of trueness that the novel tested qPCR method must meet in microbiological diagnostics. The reason concerns the uncertainty of the certified reference material to quantify the number of microorganisms of some species, being available only for viruses (e.g., HIV, HCV). For other clinically relevant microorganisms, these materials are only for qualitative evaluation [52]. For health and consumer protection, there is an accepted trueness value (within 25%) to quantify genetically modified organisms [71]. Therefore, without an accepted reference value of trueness for microalgae samples, conclusions can only be made on the influence of the matrix on the quantification and not on the actual bacterial counts.

## 5. Conclusions

The primers designed in this study, groEL200, amplified the DNA from the three tested species (*V. alginolyticus*, *L. anguillara*, and *V. harveyi*) and showed specificity when tested with DNA from other species (such as *E. coli* and *S. enterica*). This study presents quantification curves for three pathogenic species of the Vibrionaceae family using three different DNA extraction methods. The results proved that the DNeasy kit is more adequate for the qPCR analysis than the Gen kit and the boiling method since it was possible to obtain increased qPCR efficiencies. In this study, the quantification of *V. harveyi* was also attempted using a complex extraction matrix containing microalgae. The results strongly suggest that the matrices influence the qPCR efficiency, which agrees with previous studies [26,63,64,65]. Accordingly, the qPCR protocol and the standard curves presented here should only be used to quantify these species in similar prevailing conditions, such as water from aquaculture tanks. This is not a problem as it would still be useful for the early detection of pathogens and assist producers in the prevention/mitigation of vibriosis.

## Figures and Tables

**Table 1:** Species and accession numbers from NCBI used to design the primers for the *groEL* gene.

Species Strain	Accession Number
*Vibrio alginolyticus* ATCC 17749	CP006719.1
*Listonella anguillara* 775	CP002284.1
*Vibrio harveyi* FDAARGOS_107	CP014038.2
*Vibrio atlanticus* LGP32	FM954972.2
*Vibrio campbellii* ATCC BAA-1116	CP006605.1
*Vibrio cholerae* N16961	CP047297.1
*Vibrio fluvialis* ATCC 33809	CP014035.2
*Vibrio mediterranei* 117-T6	CP033577.1
*Vibrio metschnikovii* CIP 69.14	NZ_ACZO01000005.1
*Vibrio nigripulchritudo* Snf1	FO203526.1
*Vibrio parahaemolyticus* RIMD 2210633	BA000032.2
*Vibrio toranzoniae* Vb 10.8	LMXU01000002.1
*Vibrio tubiashii* ATCC 19109	CP009354.1
*Vibrio vulnificus* CECT 4999	CP014637.1
*Escherichia coli* pAR060302	HQ023864.1
*Pseudomonas aeruginosa* PAO1	CP053028.1
*Staphylococcus aureus* NCTC 8325	CP000253.1
*Salmonella enterica* CMCST_CEPR_1	CP053702.1

**Table 2:** *V. alginolyticus*, *L. anguillara*, and *V. harveyi* equations from the three DNA extraction methods (Gen kit, DNeasy kit, and boiling method). The R^2^ and the amplification factor for each method are shown as well.

DNA extraction methods	*V. alginolyticus*			
	Equation	R^2^	*E* (%)	Amplification factor
Gen kit	-------	-------	-------	-------
Boiling	------	------	------	------
DNeasy kit	y = −3.095x + 54.176	R^2^ = 0.76	110	2.10
DNA extraction methods	*L. anguillara*			
	Equation	R^2^	*E* (%)	Amplification factor
Gen kit	y = −3.288x + 49.057	R^2^ = 0.95	101	2.01
Boiling	y = −4.310x + 47.310	R^2^ = 0.97	71	1.71
DNeasy kit	y = −3.284x + 44.015	R^2^ = 0.90	102	2.02
DNA extraction methods	*V. harveyi*			
	Equation	R^2^	*E* (%)	Amplification factor
Gen kit	y = −3.296x + 45.049	R^2^ = 0.93	101	2.01
Boiling	y = −4.868x + 48.075	R^2^ = 0.94	60	1.60
DNeasy kit	y = −3.478x + 39.227	R^2^ = 0.96	94	1.94

## Data Availability

Not applicable.

## Supplementary Materials

The following supporting information can be downloaded at: https://www.mdpi.com/article/10.3390/microorganisms10102060/s1, Figure S1: Clustal W alignment using the MegAlign software (DNASTAR Inc.) for 14 species of the Vibrionaceae family and 4 outgroup species. The forward and reverse primers (groEL200) are also depicted. Grey nucleotides are a match with the consensus, whereas white nucleotides differ from the consensus. Figure S2: Example of melting curves from the three members of the Vibrionaceae family. The peaks are, respectively, 79.3 °C for *Vibrio harveyi*, 79.8 °C for *Listonella anguillara*, and 80.3 °C for *Vibrio alginolyticus.*
