# Supplementary material for: Real-Time PCR Protocol for Detection and Quantification of Three Pathogenic Members of the Vibrionaceae Family
Source: Microorganisms. 2022 Oct 18;10(10):2060. doi: 10.3390/microorganisms10102060 (PMC9609007; doi:10.3390/microorganisms10102060)
Supplement: Supplementary file 1 [file microorganisms-10-02060-s001.zip › microorganisms-1922511-supplementary.pdf]

Article

Real-time PCR protocol for detection and quantification of three pathogenic members of the Vibrionaceae family

Cátia Costa[1], Guilherme D. Ferreira[2], Marco Simões[1], Joana L. Silva[3] and Maria J. Campos [2,*]

[1] MARE-Marine and Environmental Sciences Centre, 2520-630 Peniche, Portugal
[2] MARE-Marine and Environmental Sciences Centre, ESTM, Polytechnic of Leiria, 2520-630 Peniche, Portugal
[3] Allmicroalgae Natural Products S.A., Rua 25 de Abril, s/n, 2445-413 Pataias Gare, Portugal
* Correspondence: mcampos@ipleiria.pt

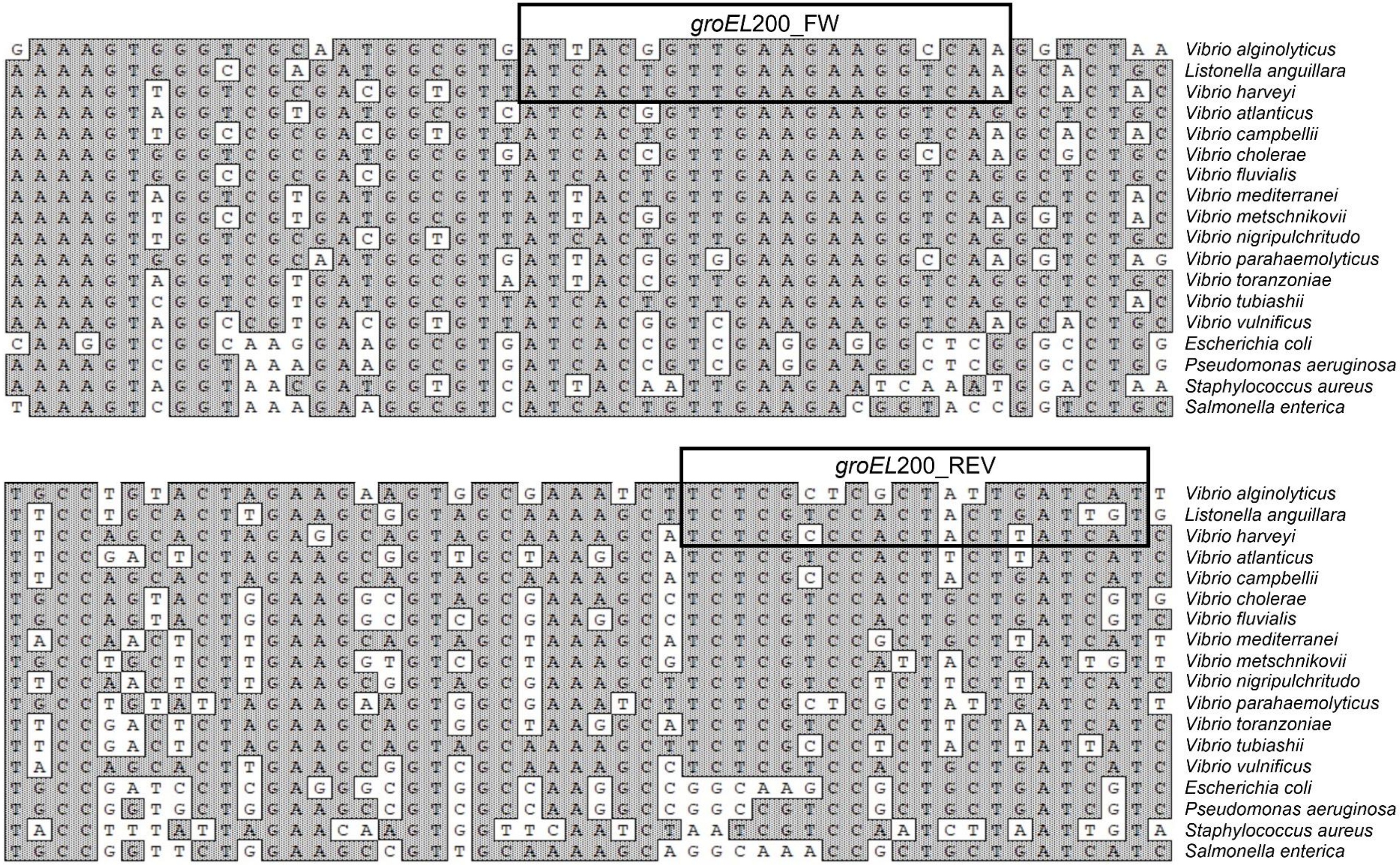

Figure S1. Clustal W alignment using the MegAlign software (DNASTAR Inc.) for 14 species of the Vibrionaceae family and 4 outgroup species. The forward and reverse primers (*groEL*200) are also depicted. Grey nucleotides are a match with the consensus, whereas white nucleotides differ from the consensus.

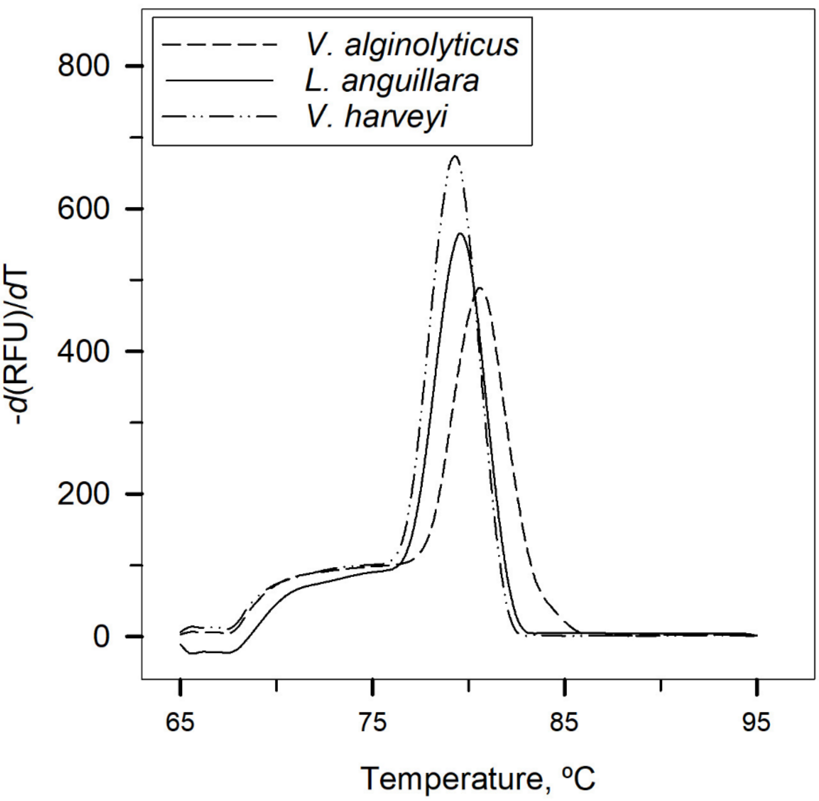

Figure S2. Example of melting curves from the three members of the Vibrionaceae family. The peaks are, respectively, 79.3 °C for *Vibrio harveyi*, 79.8 °C for *Listonella anguillara,* and 80.3 °C for *Vibrio alginolyticus.*
